# Artificial hibernation: A new technique for protecting neural tissue and organs

**DOI:** 10.4103/NRR.NRR-D-25-00025

**Published:** 2025-12-30

**Authors:** Wuhua Pang, Ziqi Wang, Yuqi Lin, Yuhan Liu, Xiaoyu Wang, Mengguang Wei, Xiaoyin Li, Xuyi Chen

**Affiliations:** 1 School of Traditional Chinese Medicine, Tianjin University of Traditional Chinese Medicine, Tianjin, China; 2 Characteristic Medical Center of Chinese People’s Armed Police Force, Tianjin, China

**Keywords:** artificial hibernation, brain hypoxia-ischemia, brain, central nervous system diseases, cerebral hemorrhage, cerebral infarction, craniocerebral trauma, hypothermia, neuroprotection, organ transplantation

## Abstract

With the rapid development of life support technologies in the biomedical field, artificial hibernation has shown significant potential in the area of neural tissues and organ protection. This paper systematically reviews the three major technologies of artificial hibernation, as well as their operational procedures and the latest technological progress. It emphasizes the protective role of artificial hibernation technology on neural tissues and vital human organs, such as the brain, spinal cord, heart, kidneys, liver, and intestines, and explores its protective mechanisms. Based on an analysis of the existing literature, it was observed that artificial hibernation technology significantly lowers metabolic rate and decelerates pathological processes, consequently enhancing organ survival rates and functional recovery. This article explains the artificial hibernation technology, neural tissue and organs, and their relationships. By discussing the protective effects of the artificial hibernation technology on neural tissue and organs, it proves the therapeutic effects of this technology in diseases such as craniocerebral injury, extensive cerebral infarction, cerebral hemorrhage, neonatal hypoxic-ischemic encephalopathy and post-cardiopulmonary resuscitation encephalopathy. Furthermore, this paper addresses the clinical challenges associated with applying artificial hibernation for neuroprotection and organ preservation, while also outlining potential future development directions for the technology. Therefore, the exploration of how artificial hibernation technology protects neural tissue and organs provides a solid theoretical foundation for its future clinical application.

**Facts**

• Neuroprotective and multi-organ protection mechanisms: The artificial hibernation technology significantly reduces damage to neural tissue and key organs (such as the brain, spinal cord, heart, kidneys, liver, and intestines) by lowering body temperature and metabolic rate. It shows potential clinical application value in the treatment of various diseases, including traumatic brain injury, extensive cerebral infarction, cerebral hemorrhage, neonatal hypoxic-ischemic encephalopathy, and post-cardiac arrest brain injury.

• Clinical translation progress: The number of clinical trials targeting artificial hibernation technology is increasing annually worldwide, particularly in the fields of traumatic brain injury, spinal cord injury, cardiac surgery, and organ transplantation. Some drugs have received conditional approval from the FDA, primarily for neuroprotection during cardiac surgery.

• Future application potential: Some drugs have received conditional approval from the FDA, primarily for neuroprotection during cardiac surgery.

**Open questions**

• How can artificial hibernation technology be further optimized to ensure its safety and efficacy in different medical scenarios, particularly regarding the long-term effects on patients under prolonged hypothermic conditions?

• What is the potential application of artificial hibernation technology in rare genetic disorders and metabolic diseases?

• What is the potential for the application of artificial hibernation technology in slowing the progression of neurodegenerative diseases?

## Introduction

### Artificial hibernation technology

As a novel medical intervention, artificial hibernation technology has attracted considerable attention in recent years. Hibernation is an inherent survival mechanism employed by some animals to endure extreme environmental conditions. Hibernating animals reduce energy consumption and lessen organ damage by decreasing their core body temperature and metabolic rate, and this condition is mainly characterized by reductions in body temperature, metabolic rate, heart rate, water loss rate, and respiratory rate (Geiser, 2013). Through hibernation mechanisms, animals’ organs and brains can effectively resist damage from severe external environments, increasing their chances of survival. For example, ground squirrels adjust to the difficulties of cold seasons and food shortages by greatly reducing their sensitivity to low temperatures and cutting down their metabolic requirements for oxygen and water (Pra et al., 2022). The main aim of artificial hibernation technology is to imitate the animal hibernation state in humans, thereby lowering metabolic rates and potentially prolonging the length of surgical operations, reducing trauma, protecting organ functions, and even maintaining life in extreme environments. The basic principle of this technology involves altering the body’s metabolic pathways and thermoregulatory systems to create a state similar to animal hibernation in humans (Logan and Storey, 2020). This state can also trigger the endogenous protective mechanisms, potentially enhancing patient survival rates and protecting crucial tissues and organs (Tarahovsky et al., 2017). Existing artificial hibernation techniques mostly include mild hypothermia, a rather well-developed method that is widely used in emergency care and surgical procedures. Newer approaches involving hibernation-inducing agents, which are convenient and effective, offer great clinical potential. Moreover, technologies using central nervous system (CNS) regulation, which allow for precise body temperature control, hold promise for future space missions and deep-sea exploration.

### Concepts and classifications of neural tissues and organs

Neural tissue is the main component of the nervous system, and it is responsible for receiving, processing, and transmitting information. Neural tissue mainly consists of two main types of cells: neurons and glial cells (Dhureja et al., 2025). Neurons form the basic functional elements of the nervous system, and they possess the ability to receive stimuli, transmit impulses, and produce responses. They can be divided into different types on the basis of their morphological features and functional tasks, such as sensory neurons, motor neurons, and interneurons. Sensory neurons are of great significance in converting external physical and chemical stimuli into neural signals, which are then sent to the CNS (Antonioni et al., 2024). Motor neurons, in contrast, carry instructions from the CNS to muscles and glands to yield appropriate motor responses. Interneurons are located between sensory and motor neurons and play an important part in the integration and transfer of neural information. Glial cells perform vital supportive, protective, and nourishing functions in neural tissues, and these cells include astrocytes, oligodendrocytes, and microglia. Astrocytes, the largest glial cells, are crucial in maintaining the neuronal microenvironment by providing necessary nutrients and getting rid of metabolic waste products. Oligodendrocytes play an important role in forming the myelin sheath around neurons, which greatly increases the conduction speed of nerve impulses. Microglia serve as the chief immune cells in the nervous system and perform a very important role in clearing away damaged or dead neurons (Ju and Hang, 2024). Neural tissue can also be classified on the basis of its functional and anatomical traits. For example, depending on its function, neural tissue can be classified into CNS tissue and peripheral nervous system (PNS) tissue. The CNS consists of the brain and spinal cord and is of key importance in processing and integrating information (Cai et al., 2025). The PNS consists of the cranial and spinal nerves and plays an important role in transmitting information to various parts of the body.

The neural organs, which are the specialized structures that constitute the nervous system, mainly include the brain, spinal cord, and peripheral nerves (Varadarajan et al., 2022). The brain is the top -level command center of the nervous system and consists of important parts such as the cerebral cortex, basal ganglia, brainstem, and cerebellum. The cerebral cortex holds great importance in controlling higher-level cognitive functions such as memory processing, language understanding and generation, and decision-making, while the basal ganglia are of vital importance in motor control and emotional regulation. The brainstem is crucial for regulating essential life -sustaining activities, including respiration and heart function, and the cerebellum is very important for coordinating movements and maintaining balance. The spinal cord, an essential part of the CNS that is located inside the vertebral column, acts as a pathway for transmission of information between the brain and different areas of the body (Saade and Marti, 2025). The spinal cord is made up of gray matter and white matter. Gray matter mostly contains neuronal cell bodies and is in charge of information processing, while white matter, which is mainly composed of nerve fibers, plays a key role in promoting the transmission of neural information. The PNS serves as a vital connection linking the CNS to various parts of the body (Tsuchiya et al., 2025). Twelve pairs of cranial nerves are responsible for the sensory and motor functions of the head and face, while spinal nerves derived from the spinal cord are crucial for transmitting sensory information and enabling motor control in different regions of the body. Peripheral nerves can also be classified into sensory, motor, and mixed nerves according to their functional features. Sensory nerves are in charge of carrying sensory information from various areas of the body to the CNS; motor nerves play a very important role in sending instructions from the CNS to different parts of the body; and mixed nerves perform both sensory and motor functions (Milosevic et al., 2020).

### Basic concepts and classification of other organs

The heart is the main organ responsible for pumping blood in the human body and performs the crucial task of circulating blood throughout the body. The heart is made up of cardiac muscle tissue, which shows the characteristics of automatic rhythm and electrical conduction. The heart can be anatomically and functionally divided into the left and right atria as well as the left and right ventricles, with the atria mainly being in charge of receiving blood and the ventricles responsible for pumping blood out of the heart (Neradilova et al., 2024). The kidneys are very important excretory organs in the human body and are of great importance in filtering waste products and excess solutes from the blood, forming urine and then expelling it from the body. Nephrons are the structural units of the kidney, with each nephron containing a glomerulus and a renal tubule in charge of filtration and reabsorption (Trask-Marino et al., 2025). The liver is the chief metabolic organ in the human body and is of great importance in the synthesis, breakdown, and storage of various substances. The liver consists of hepatocytes, which perform a large number of essential functions such as protein synthesis, bile generation, and detoxification (de Hoyos-Vega et al., 2021). The intestine is an important digestive organ in the human body and plays a key part in the digestion and absorption of nutrients from food (Galsgaard et al., 2025). The digestive system is made up of the gastrointestinal tract and related digestive glands. The gastrointestinal tract includes the mouth, esophagus, stomach, small intestine, and large intestine, which are highly important for both mechanical and chemical digestion of eaten food, and the digestive glands, which secrete digestive enzymes and fluids that are involved in the chemical digestion of nutrients (Calamita and Delporte, 2023).

### Correlations of artificial hibernation technology with neural tissues and multiple organ systems

Artificial hibernation technology is a therapeutic approach that can reduce neural harm by reducing the body temperature and metabolic rate to substantially cut down the energy usage and metabolic requirements of neural tissues. This approach can protect neural tissue from ischemic and hypoxic damage by restraining neuronal activity, thereby making nerve cells less excitable, and weakening synaptic transmission. Moreover, a low-temperature setting has been shown to ease neuroinflammation and oxidative stress, offering more protection for neuron structure and function. Thus, artificial hibernation technology shows great potential for clinical use in neuroprotection, especially in handling brain injuries and neurodegenerative disorders. This technology has shown remarkable effects on neural organs such as the brain and spinal cord: It can considerably reduce brain metabolic activity when body temperature is lowered, lessening cerebral blood flow and mitigating brain swelling and elevated intracranial pressure (ICP). It can also effectively protect the spinal cord from harm during spinal surgeries and spinal cord injuries (Li et al., 2025) and help manage epilepsy and neuralgia by regulating neurotransmitter release and reducing excessive nervous system excitement (Rahman et al., 2024). Thus, this approach shows a deep-seated influence on the nervous system and neural organs. Artificial hibernation technology can also play a crucial role in protecting other vital organs such as the heart, kidneys, liver, and intestines. The reduced body temperature can reduce the metabolic load on the cardiovascular system, resulting in a slower heart rate and lower blood pressure and thus maintaining cardiac function. In the kidneys, hypothermia can reduce renal tubular damage and preserve kidney function (Ichimata et al., 2024). In the liver, artificial hibernation can decrease the metabolic demands on liver cells and protect the liver’s detoxification ability (Suita et al., 2023). Finally, for the intestines, lower temperatures can reduce intestinal movement and decrease digestive juice secretion, thereby cutting down the risk of intestinal injury (Faingold et al., 2016).

At present, artificial hibernation technology is being applied in disease therapy and surgical operations and can be efficiently used for preservation of neural tissues and organs (Werner et al., 2024). When dealing with diseases and surgeries, medical professionals often face difficulties in addressing the risk factors that can cause secondary injuries, wherein malfunction of one organ could have negative effects on several organ systems (Calabrese et al., 2023). Therefore, the task of protecting neural tissues and organs has become a crucial focus in modern biomedicine, and the use of artificial hibernation technology may facilitate neural tissue and organ protection, decrease surgical risks, and prolong patient survival. In the induced hibernation state, patients with serious conditions such as stroke (Zheng et al., 2024), heart disease, and trauma (Tarahovsky et al., 2017) show significantly enhanced recovery abilities. In organ transplant surgery, immune rejection remains an unavoidable problem (Platt and Cascalho, 2023), and the preservation of neural tissues and organs is equally important. Artificial hibernation technology can play an important role in organ transplant surgery by lengthening the duration of organ preservation and lessening immune rejection reactions. This article explores the mechanisms, applications, and existing understanding of the protective effects of artificial hibernation technology on neural tissue and organs.

This review aims to systematically analyze the core mechanisms by which artificial hibernation technology regulates neural and multi-organ protection. It also aims to explore the potential of this technology to increase the time window for treating ischemic injuries, optimize the preservation of organs during transplantation, and improve critical care strategies. Finally, this paper provides an innovative theoretical basis for clinical responses to major challenges such as stroke, myocardial infarction, and organ shortage.

## Search Strategy

We used the PubMed database for online search using the keywords “artificial hibernation” and “neuroprotection.” The time for the search was between February 1962 and February 2025. More thorough screening was carried out according to the titles and abstracts and studies which showed little relevance to the research topic were removed, and thus only 150 studies that completely satisfied the pre -set inclusion criteria were finally kept.

## Classification and Recent Technological Advancements in Artificial Hibernation Technology

The artificial hibernation approach can be traced back to medical operations in the mid-20^th^ century, when a mixture of pharmacological substances was utilized to decrease patients’ metabolic rates in reaction to severe infections or injuries (Veghelyi, 1962). At present, artificial hibernation involves three main approaches, namely, mild hypothermia therapy, induction of artificial hibernation, and CNS modulation to induce hibernation. Moreover, depending on the core body temperatures, these approaches can be further divided into ultra-deep hypothermia (less than 17°C), deep hypothermia (17–27°C), moderate hypothermia (28–32°C), and mild hypothermia (33–35°C) (Drewry and Mohr, 2022). Mild hypothermia treatment mainly involves achieving a considerable decrease in core body temperature through both noninvasive and invasive physical cooling methods along with administration of centrally acting anesthetic drugs such as sedative anesthetics and combinations of drugs containing pethidine, chlorpromazine, and promethazine (Sessler, 2009). Research on induction of artificial hibernation has identified specific substances in the bodily fluids of hibernating mammals that regulate core body temperature and metabolic rate by activating brain areas related to hibernation (Ichinose and Hindle, 2024). This finding was confirmed in subsequent experiments where researchers injected plasma factors drawn from hibernating groundhogs into non-hibernating ground squirrels, which induced a hibernation state in the squirrels (Feketa et al., 2023). Several compounds that can potentially simulate hibernation have been identified, including 5′-adenosine monophosphate and hydrogen sulfide. CNS regulation technology involves inducing a hibernation-like state through targeted modulation of certain neural cell groups within the CNS (Yang et al., 2023a, b). The hypothalamus acts as a key connection point between the nervous and endocrine systems, controlling most of the autonomic nervous reactions, including metabolism, circadian rhythms, and sleep. Various nuclei inside the hypothalamus play important roles in regulating hibernation (Zhou et al., 2023). Transcranial ultrasound stimulation of the preoptic region of the hypothalamus has been shown to induce hibernation in mice, and this technique has been used to adjust body temperature and metabolic rate and trigger protective mechanisms, offering new possibilities for future medical uses.

**Figure 1:**
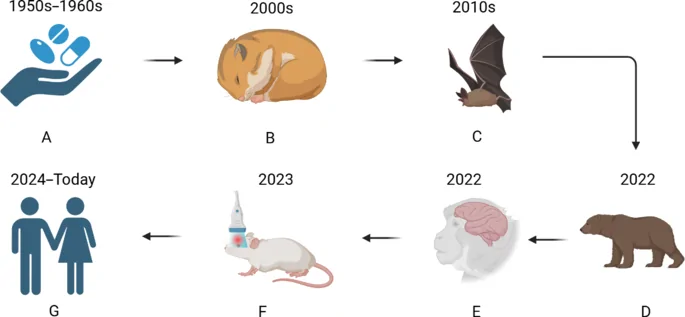
Key milestones in the development of artificial hibernation. (A) In 1951, French doctors Laborit and Huguenard firstly applied chlorpromazine (the core ingredient of hibernation mixture) to the clinic, through the combination of drugs to inhibit the central nervous system and induce patients to enter a low temperature metabolic state (Kunz, 2014). (B) In 2006, scientists found circannual control of hibernation by HP complex in the brain (Kondo et al., 2006). (C) In 2014, scientists proposed the hormone regulation hypothesis of bat hibernation (Willis and Wilcox, 2014). (D) In 2022, scientists clarified the cortisol-metabolism coupling mechanism in bears (Frobert et al., 2023). (E) In 2022, the Chinese team achieved stable hypothermia of central nervous regulation in non-human primates (macaques) and mapped a whole-brain neural network for thermoregulation (Zhang et al., 2023). (F) In 2023, a team from the University of Washington in the United States developed ultrasound induced hypothermia and hypometabolism technology (UIH), which enables mice to enter a continuous hibernation state by non-invasionally stimulating the preoptic region of the hypothalamus (Yang et al., 2023a, b). (G) The technology is expanding into clinical and aerospace fields, with the goal of achieving controlled and safe human hibernation (Shi et al., 2021).

The operational approaches of the three technologies vary considerably. At present, low-temperature technology has been shown to lessen the body’s reaction to pathological stimuli and cut down the basal metabolic rate, presenting a novel therapeutic approach for safeguarding organs. Although low-temperature technology is relatively advanced and has been utilized in intensive care units, the associated apparatus is large and the process involves intricate protocols that require extra technical assistance (Drewry and Mohr, 2022). Moreover, long-term exposure to sub-therapeutic hypothermia could result in a host of complications, dependency inclinations, and drug resistance. Therefore, low-temperature technology needs to be advanced further. Techniques involving artificial hibernation inducers are also fairly developed. Although relevant inducers capable of making animals such as mice enter a state resembling hibernation are available, the types of inducers available at present are limited, and their mechanism of action is still not entirely clear. Thus, in the future, more extensive, secure, and appropriate inducers for humans should be explored to boost the application of artificial hibernation technology. In contrast to the other two approaches, the technology for CNS regulation to induce hibernation is rather underdeveloped. Research on this technology is presently concentrated on brain region mechanisms in mice and rats, but this area holds great potential for growth (Wang et al., 2024). Artificial hibernation technology still requires substantial improvement and refinement in terms of technology and safety, including aspects such as the precision of controlling the hibernation state, protecting the well-being of patients or astronauts, and resolving recovery issues after the hibernation state. These crucial technical challenges have to be addressed in future studies. A comparative analysis with the merits and demerits of each approach is summarized in **Table 1**, and the key stages in the development of artificial hibernation technology are depicted in **Figure 1**.

**Table 1:** Analysis of three artificial hibernation technologies

Artificial hibernation method	Intervention measure	Advantage	Drawback
Low temperature technology	Physical cooling; chemical cooling; central nervous system anesthetics	Relatively mature technology; reduces response to pathological stimuli; reduces basal metabolic rate	Large equipment size; complexity of implementation; addictive, resistant; toxic side effects
Artificial hibernation inducer technology	Natural compound synthetic inducers	Moderate technology maturity; ease of access	Fewer types of inducers; mechanism of action not fully understood
Central nervous system modulation technology	Electricity, magnetism, ultrasound, light, chemical genetics	High precision; short cooling time; sustainability	Lower technological maturity; requires high-precision equipment and advanced technical support

This table compares the intervention measures, advantages, and drawbacks of three types of artificial hibernation methods, presenting the development levels of these methods in an intuitive manner.

## Protective Mechanisms Associated with Artificial Hibernation Technology

### Mechanisms underlying the protective effects of artificial hibernation technology on the brain and spinal cord

Traumatic brain injury (TBI) is a complex pathologic process affecting both the white and gray matter of the brain. Injury to the white matter can set off a severe chain reaction involving vascular leakage, edema, and secondary demyelination, resulting in long-term functional disorders such as cognitive decline and motor disorders (Bai et al., 2020; Yang et al., 2025; You et al., 2025). Moreover, the damaged myelin sheath is engulfed by macrophages, and inflammatory mediators are released during the engulfment process (Zhou et al., 2019). Artificial hibernation can protect the myelin sheaths by retarding the macrophage-mediated engulfment of the damaged myelin and stopping the excess release of inflammatory mediators due to overactivation of macrophages, thereby promoting nerve repair. Conversely, gray matter damage leads to neuronal functional alterations such as cognitive dysfunction, mood changes, and local metabolic anomalies (Packer et al., 2024).

Research on artificial hibernation technology for brain protection has shown that a low-temperature state lessens the brain’s need for oxygen, slows down cellular metabolism, and reduces energy expenditure and the production of harmful free radicals, thus protecting brain cells. Takahashi et al. (2020) successfully induced hibernation in rodents by stimulating hypothalamic neural circuits using optogenetics, which resulted in a decrease in body temperature and oxygen consumption. Importantly, the animals still had the ability to control their metabolism and showed no serious tissue or organ damage or functional problems after they recovered. These results confirm the crucial role of the hypothalamic neural circuits in regulating hibernation and emphasized the potential of hibernation therapy for protecting the brain. During an acute brain injury, injured cells release a range of inflammatory substances that worsen neuronal damage, increase the likelihood of cerebral vasospasm, and enhance brain edema and oxidative stress to eventually exacerbate tissue injury (Schneider et al., 2018). However, artificial hibernation reduces secondary injury by preventing inflammatory reactions and decreasing the release of inflammatory substances (Liu et al., 2024a, b). Eskla et al. (2018) conducted a study on human cervical cancer HeLa cells and mouse embryonic fibroblasts and showed that low temperature induces large-scale changes in gene expression and turns on multiple transcription factors related to the antioxidant system and hypoxia response pathways, such as nuclear factor erythroid 2-related factor 2 (Nrf2) and hypoxia-inducible factor-1α. This process causes the upregulation of antioxidant genes such as Gclc and Trxr1, elevating intracellular glutathione levels and strengthening the cell’s resistance to oxidative stress. Moreover, low temperature strengthens hypoxia-inducible factor-1α activity, speeding up metabolic adaptation to hypoxia and showing protective effects on brain tissue. Moreover, during brain injury, buildup of neurotransmitters such as glutamate may cause excessive excitement and then damage neuronal cells. Li et al. (2024) used noninvasive transcranial macroscopic near-infrared-II fluorescence imaging to prove that hypothermia could boost lymphatic inflow by slowing down the flow rate of cerebrospinal fluid. In addition to increasing lymphatic inflow, hypothermia can speed up toxin metabolism in the brain and reduce the spread of inflammatory responses, thus protecting brain tissue. Zhang et al. (2025b) conducted a series of experiments using a rat model of acute ischemic stroke to explore how the combination of hypothermia and MgSO_4_ treatment affected various neurovascular cells after oxygen-glucose deprivation/reoxygenation injury, and their results showed that the combined treatment was much more effective than either hypothermia or MgSO_4_ treatment alone. Thus, the combination of hypothermia and MgSO_4_ could protect different neurovascular cells by adjusting the intracellular Ca^2+^ balance. Zhang et al. (2025a) also used rat models of cerebral ischemia-reperfusion and *in vitro* experiments to demonstrate that mild hypothermia (MH) reduced oxidative stress and cell apoptosis and improved neural damage by lowering the expression of S100A8 protein, activating the calcium/calmodulin-dependent protein kinase kinase 2 and AMP-activated protein kinase signaling pathways, and enhancing mitochondrial function. Overexpression of S100A8 counteracted the neuroprotective effects of MH while its knockdown imitated these protective effects.

Spinal cord injury (SCI) is an extraordinarily intricate pathological process with complicated and varied negative outcomes, and recent research has shown that it affects numerous aspects such as immunity (Nielsen et al., 2024), blood vessels, lipid and blood sugar metabolism, bone health, autonomic nerve function, and psychology (Noonan, 2023). Research on the neuroprotective effects of artificial hibernation technology against SCI has indicated that this method significantly lowers the metabolic rate of spinal cord neurons, reducing the oxygen and nutrient requirements. Moreover, the anti-inflammatory effects of artificial hibernation technology for SCI mainly manifest in restraining the release of inflammatory factors, cutting down oxidative stress, and regulating immune cell function. In contrast, the anti-edema mechanism chiefly involves reducing capillary permeability, enhancing microcirculation, and decreasing cerebrospinal fluid production, consequently strengthening cellular tolerance to ischemic and hypoxic situations (Quinones et al., 2024). Zhang et al. (2023) conducted primate experiments in which they successfully achieved a steady drop in body temperature by activating neurons in the preoptic area of the hypothalamus, thus protecting tissues from cold-induced damage. Artificial hibernation was shown to reduce the buildup of metabolic byproducts in neuronal cells and diminish the risk of cellular harm (Wang et al., 2024). After SCI, the body generates a large quantity of inflammatory mediators, such as interleukin (IL)-1β, IL-6, and tumor necrosis factor (TNF)-α. These inflammatory factors tend to worsen tissue damage. Dos and Park (2021) proved that therapeutic hypothermia (TH) could considerably cut down the release of IL-1, IL-6, and TNF-α at both serum and tissue levels, thus alleviating secondary injury. Artificial hibernation technology can reduce the levels of inflammatory factors, block apoptosis signal transmission, and reduce the expression of axonal growth-inhibiting factors. Therefore, it has the potential to ease the inflammatory response and lessen nerve damage after SCI. Fu et al. (2022) showed that MH treatment regulated microglial M1/M2 polarization by inhibiting the Toll-like receptor 4/nuclear factor (NF)-κB signaling pathway, thus repressing microglial activation and M1 polarization while lowering inflammatory factor levels. Moreover, this treatment promoted M2 polarization of microglia, mitigated inflammatory responses, protected spinal cord neurons, and enhanced the motor function in a rat model of SCI. Artificial hibernation also set up an immunosuppressive and oxidative defense mechanism by modifying neutrophil function and white blood cell counts and raising antioxidant levels, which effectively reduced the risk of reperfusion injury (Liu et al., 2024a, b). Oxidative stress after SCI can lead to increased intracellular levels of reactive oxygen species, harming the structural soundness and functional ability of spinal cord tissue (Xu et al., 2014). Fu et al. (2023) successfully used the A1 adenosine receptor agonist N6-cyclohexyladenosine to induce a hibernation-like state that closely resembled physiological hibernation in mice, and this intervention effectively eased LPS-induced neuroinflammation and maintained the integrity of the blood–brain barrier (BBB). Moreover, by restraining pro-inflammatory reactions in macrophages and lessening oxidative stress damage in endothelial cells during systemic hypothermia, their method protected spinal cord tissue, providing strong evidence for the potential of artificial hibernation as a therapeutic approach to protect spinal cord tissue. Ahn et al. (2023) used a rat model of ischemia-reperfusion injury (IRI) to demonstrate that hypothermia treatment after cardiac arrest and the return of spontaneous circulation could protect motor neurons in the lumbar spinal cord by activating the Nrf2/heme oxygenase (HO)-1 signaling pathway and alleviating IL-1β-induced inflammation and reactive astrocytosis, thereby improving hind limb paralysis caused by cardiac arrest. This finding highlighted the possible benefits of TH for spinal cord protection. Moreover, during hibernation, the normal operation of calcium-binding proteins regulates Ca^2+^ transients and contributes to maintaining internal environmental stability, thereby safeguarding the functional integrity of nerve cells (Gattoni and Bernocchi, 2019).

**Figure 2:**
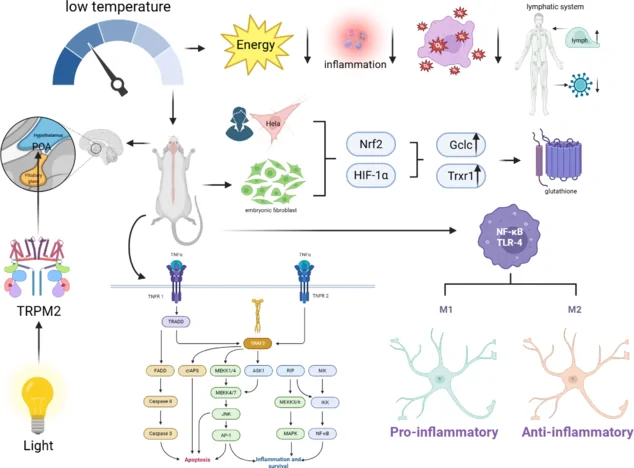
Mechanism of artificial hibernation in protecting the central nervous system. Created with BioRender.com. Gclc: Glutamate-cysteine ligase catalytic subunit; HIF-1α: hypoxia-inducible factor 1-alpha; NF-κB: nuclear factor kappa B; Nrf2: nuclear factor erythroid 2-related factor 2; POA: primary optical area; TLR-4: toll-like receptor 4; TNFR1: tumor necrosis factor receptor 1; TNFα: tumor necrosis factor-alpha; Trxr1: thioredoxin reductase 1.

In conclusion, artificial hibernation technology can play a vital role in protecting the spinal cord through mechanisms such as decreasing cellular oxygen demand and lessening inflammatory reactions, which are summarized in **Figure 2**.

### Mechanisms underlying the protective effects of artificial hibernation technology on cardiac function

Under artificial hibernation, the oxygen consumption of myocardial cells is substantially reduced (Carulli et al., 2025). This reduction eases the cardiac load and lowers the risk of myocardial ischemia and infarction by decreasing the heart’s contraction and relaxation rates, as well as its heart rate, thereby promoting the recovery of cardiac tissue (Skrifvars and Abella, 2024). Vaidya et al. (2023) found that in stunned myocardium, coronary blood flow at rest decreased while myocardial oxygen requirements increased, ultimately leading to weakened cardiac contractility. Thus, artificial hibernation can increase coronary blood flow or decrease myocardial oxygen demand, potentially restoring the contractile function of injured myocardium, thereby providing myocardial protection. Ahn et al. (2023) conducted a proteomics study on the cardiac protection mechanisms in American marmots during hibernation. Their findings revealed substantial changes in the protein network related to cardiac protection, including the upregulation of antioxidant enzymes like catalase, downregulation of endoplasmic reticulum stress-response proteins such as GRP78, activation of nitric oxide signaling pathways, acute-phase responses, and transcriptional regulation by CREB and NFAT. They also noted the involvement of protein kinase A and α-adrenergic signaling pathways. Yang et al. (2021) demonstrated that the signal transduction efficiency of L-type calcium channels (LCCs) and ryanodine receptors was significantly enhanced during hibernation in ground squirrels. Despite a decrease in LCC expression in myocardial cells, the unexpected increase in the efficiency of Ca^2+^ signal transduction helped maintain cardiac contractile function. This enhancement was attributed to the upregulation of junctophilin-2 and caveolin-3, which strengthened the transverse tubule-sarcoplasmic reticulum junction and improved LCC-ryanodine receptor coupling. This enhanced calcium signaling during hibernation not only protected the myocardium but may also have therapeutic potential for reversing heart failure through activation of the myocardin-serum response factor pathway. In a subsequent study, Yang et al. (2023) conducted a thorough comparison of the transcriptomic differences between hibernating and non-hibernating ground squirrels. Their results indicate significant changes during hibernation, including cytoskeletal protein remodeling, decreased protein synthesis, and downregulation of the ubiquitin-proteasome pathway. Metabolomics analysis revealed an increase in free amino acid levels, activation of the glutathione antioxidant system, a shift in myocardial fatty acid metabolism, and enhanced activity of the pentose phosphate pathway. These findings provide new insights into cardiac protection during hibernation from the perspectives of both gene expression and metabolites, highlighting the protective effects of hibernation on the heart. Artificial hibernation may also protect the cardiovascular system by regulating inflammatory responses. Aggarwal et al. (2022) found that the expression of peroxisome proliferator-activated receptor gamma coactivator-1α, a key regulator of mitochondrial energy metabolism and an inhibitor of oxidant-stress-inflammatory signaling, was downregulated in hibernating myocardium. When hibernation is induced, the drop in body temperature can reduce the release of inflammatory mediators, thereby alleviating the cardiovascular damage caused by inflammatory responses.

After a myocardial infarction, patients undergoing cardiovascular surgery are more prone to experience cardiogenic shock, which has a mortality rate of nearly 50%. However, TH has shown numerous positive physiological effects in patients with cardiogenic shock, including improvements in cardiac function after ischemia, regulation of hemodynamic parameters, and a reduction in myocardial injury (Llerena-Velastegui et al., 2024). Moreover, it may reduce the risk of end-organ damage caused by long-term insufficient perfusion, and induced hibernation is expected to become a promising treatment method for cardiogenic shock following myocardial infarction. In comparison with normal temperature, mild TH (mTH) can provide better results in maintaining blood pressure, safeguarding organs, and alleviating oxidative stress after cardiac arrest. mTH can enhance the recovery rate of the circulatory system and decrease the mortality rate in patients experiencing cardiac arrest due to ventricular fibrillation. Gotberg et al. (2010) evaluated findings for 20 patients with acute myocardial infarction who received percutaneous coronary intervention (PCI), and their results suggested that the combination of hypothermia and adjuvant therapy may reduce the infarct area measured by magnetic resonance imaging (MRI) 3 days after the procedure. Specifically, the standardized myocardial infarction area in the hypothermia group decreased by 38%, and both the peak and cumulative release of troponin T in this group dropped significantly. In the study by El et al. (2021), among 50 patients with anterior ST-segment elevation myocardial infarction (STEMI) undergoing PCI, no patients in the intracoronary hypothermia group experienced atrial fibrillation, while three patients in the traditional treatment group did. This implies that selective intracoronary hypothermia during PCI for anterior STEMI could be safely incorporated into standard PCI procedures and was effective in reducing myocardial reperfusion injury.

**Figure 3:**
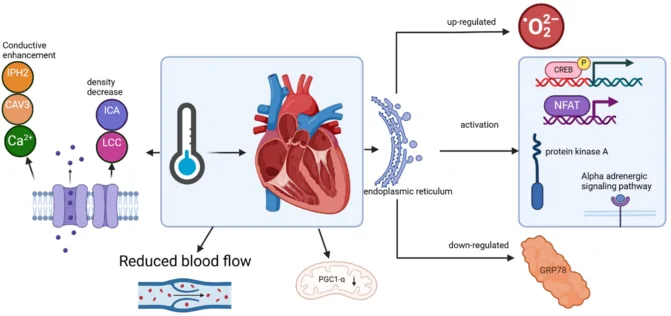
Schematic illustration of the cardioprotective mechanism induced by artificial hibernation. Created with BioRender.com. CAV3: Caveolin-3; CREB: cAMP-response element binding protein; GRP78: glucose-regulated protein 78; LCC: L -type calcium channels; IPH2: isocitrate dehydrogenase 2; PGC-1α: peroxisome proliferator-activated receptor gamma coactivator 1-alpha.

In brief, artificial hibernation has a vital protective effect on the heart since it reduces myocardial oxygen consumption, regulates the efficacy of Ca^2+^ transport, and shows other beneficial effects. The specific protective mechanisms are shown in **Figure 3**.

### Mechanisms underlying the protective effects of artificial hibernation technology on the kidney, liver, and intestine

In the state of induced hibernation, renal metabolic activity shows a remarkable slowdown, significantly reducing the energy and oxygen needs of the organ, lowering the kidney’s requirement for crucial nutrients, and decreasing the buildup of metabolic waste products. Kulthinee et al. (2024) found that during hibernation, the drop in mean arterial pressure, along with an increase in sympathetic nerve activity, stimulated the adrenal zona glomerulosa and juxtaglomerular cells, leading to an increase in plasma renin activity and aldosterone levels. Furthermore, alterations in the ultrastructure of adrenal zona glomerulosa cells indicated enhanced adrenal steroid production, which promoted sodium reabsorption in the renal tubules to maintain stable blood volume and pressure, thus providing protection to the kidneys. Renal IRI is a major cause of acute kidney injury and increases the risk of chronic kidney disease. In their mouse study, Schleef et al. (2022) demonstrated that mTH at 34°C during ischemia effectively preserved renal function and structure, preventing IRI. Additionally, mTH alleviated renal dysfunction by maintaining mitochondrial integrity and regulating systemic and local inflammatory responses during the acute phase. These protective effects not only appeared during the acute phase but also significantly reduced long-term renal atrophy and fibrosis, as well as chronic renal inflammation. In their study on renal IRI caused by asphyxia-induced cardiac arrest in male rats, Jawad et al. (2021) reported that TH significantly mitigated histopathological changes in post-ischemic renal tissue and reduced blood urea nitrogen, serum creatinine, and malondialdehyde levels in renal tissue. Moreover, TH diminished renal injury and increased the expression of Nrf2 and heme oxygenase 1 over time. Yamamoto et al. (2020) established that adenosine triphosphate (ATP) plays a vital role in renal metabolism, with its deficiency in renal tubular cells closely linked to the development of kidney diseases. During the acute stage of post-thrombotic syndrome, the recovery of ATP levels in renal tubular cells was severely delayed, leading to worsened renal fibrosis. However, under hypothermic conditions, the rate of ATP recovery in these cells was significantly faster, resulting in a decrease in fibrosis, thus strongly supporting the use of hypothermia as a protective strategy for renal tissue.

Under artificial hibernation conditions, the metabolic rates of hepatic and intestinal cells decrease considerably, leading to lessened energy requirements (Zhang et al., 2024a, b). Since the liver functions as the metabolic hub of the human body, it plays an important role in the synthesis and detoxification of macromolecules and supplies essential energy to the organs scuh as the brain and muscles. Kurtz et al. (2021) stated that extended fasting during hibernation greatly slowed down intestinal peristalsis and lessened the functional demands on the digestive system, making the intestines less sensitive to external stimuli, reducing the reliance on nutrient intake, and thereby decreasing the risk of metabolic-byproduct buildup and cellular harm. Alva et al. (2018) reported that, in comparison with mice, Syrian hamsters showed greater resistance to peroxidation in liver phospholipid tissue, which enabled effective prevention of lipid peroxidation and ferroptosis when dietary α-tocopherol was utilized and thereby strengthened cold resistance in the liver. A drop in the body temperature may also help slow down the progression of liver damage induced by ischemia. Otis et al. (2017) showed that the livers of hibernating squirrels were more resistant to injury caused by *in vitro* cold IR, and after thermal IR, the increase in the plasma levels of liver enzymes (alanine transaminase and aspartate aminotransferase [AST]) and the apoptotic index of hepatocytes were much lower in hibernating squirrels than in spring squirrels, indicating a protective influence of hibernation on liver function. Mesenteric IRI can lead to systemic inflammation and multi-organ dysfunction. Santora et al. (2010) indicated that artificial hibernation could protect distant organs and selectively modify the IRI-activated transcriptome in the lungs during superior mesenteric artery occlusion in rats, offering new perspectives for potential diagnostic and therapeutic targets for mesenteric IRI. Moreover, Kurtz et al. (2021) demonstrated that hibernation protected the organism from nutritional and oxygen variations during fasting and arousal cycles by adjusting the intestinal epithelium, immune reactions, and cell survival pathways in the gut. The liver and intestine jointly regulated lipid metabolism and systemic cholesterol dynamics during fasting while also determining the composition and variety of the intestinal microbiota, thus affecting metabolite generation. Gao et al. (2024) proved that the gut microbiota play a vital role in animal nutrition and health and contribute greatly to the maintenance of energy metabolism and intestinal immune function during hibernation, and the expression of Toll-like receptor 5 (TLR5) was upregulated at this time. TLR5 recognizes bacterial flagellin, which activates the NF-kB signaling pathway to initiate an anti-inflammatory response. This response strengthens the defensive abilities of intestinal epithelial cells and maintains the integrity of the intestinal barrier, safeguarding the intestine from possible damage. For digestive system surgeries, Goncalves-Ferri et al. (2022) demonstrated that the application of mTH during modified Bell stage II/III treatment for necrotizing enterocolitis in preterm infants was highly effective, and in their study involving 43 preterm infants with this condition, those in the mTH group needed fewer surgical interventions, showed a lower occurrence of intestinal perforation, endured a shorter duration of parenteral nutrition, and did not require extensive intestinal resections. Moreover, mTH significantly reduced the need for surgical intervention, shortened the length of parenteral nutrition, and lessened the mortality risk. The protective effect of artificial hibernation technology on hepatic and intestinal cells also considerably improved the prognosis for critically ill patients with liver and intestinal diseases.

**Figure 4:**
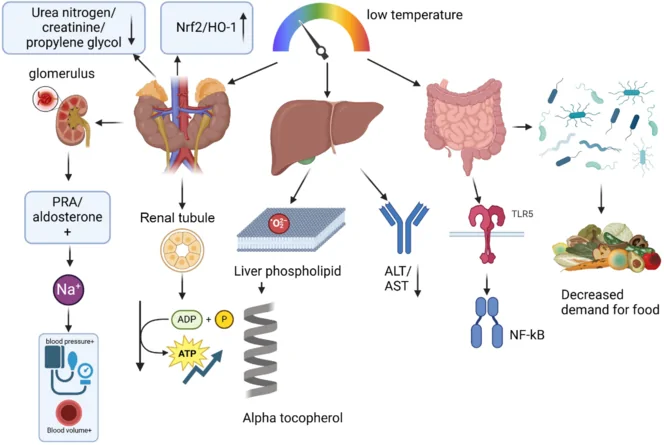
Schematic illustration of the protective mechanisms conferred by artificial hibernation on renal, hepatic, and intestinal tissues. Created with BioRender.com. ADP: Adenosine diphosphate; ALT: alanine aminotransferase; AST: aspartate aminotransferase; ATP: adenosine triphosphate; HO-1: heme oxygenase 1; NF-κB: nuclear factor kappa B; Nrf2: nuclear factor erythroid; PRA: panel reactive antibody; TLR5: toll-like receptor 5.

In brief, artificial hibernation technology protected crucial organs like the kidneys, liver, and intestines by reducing their energy and oxygen needs, adjusting metabolic rates, and other mechanisms. The detailed procedure is shown in **Figure 4**.

### Protective efficacy of artificial hibernation technology in safeguarding other organs and enhancing organ transplantation outcomes

#### Safeguarding other organs

Cooling methods linked to artificially induced hibernation can also be effective in skin-related disorders. Kang et al. (2024) conducted a thorough assessment of the effectiveness of scalp cooling in reducing chemotherapy-induced hair loss after 6 months of continuous chemotherapy. Their results showed that at 6 months, the rate of hair loss was 13.5% in the scalp cooling group and 52.0% in the traditional treatment group, and the average difference in hair thickness alteration from the starting point between the two groups was 9.0 μm, while the average difference in hair density alteration was –3.3 hairs/cm^2^. These findings indicated that scalp cooling significantly decreased the occurrence of chemotherapy-induced hair loss over a six-month span in comparison with the traditional treatment group. In a study on eye-related diseases, Qi et al. (2023) examined 58 patients with crossed eyes who had their lateral rectus muscles recessed under general anesthesia. Among the 29 patients in the ice mud treatment group, 62.5% experienced the oculocardiac reflex during surgery, as opposed to 96.6% of the 29 patients in the control group. This finding suggested that the local cooling effect induced by ice mud is a potentially easily accessible method for reducing the occurrence of the oculocardiac reflex and thus making strabismus surgery safer. In general, artificial hibernation technology, which changes both the internal and external environments of the body, has shown promise in safeguarding numerous organs.

#### Organ transplant

In terms of both quality of life and life expectancy, organ transplantation is the best alternative treatment for patients with late-stage organ failure (Lepoittevin et al., 2022). In organ transplantation procedures, the preservation of organs is a crucial step before the transplantation operation. Static cold storage is currently the predominant method for organ preservation, but the refrigeration process can lead to ischemic harm. Artificial hibernation technology allows preservation and transport of donated organs in a controlled low-temperature state with minimal metabolic activity after donation (Malinoski et al., 2023). This can greatly extend the preservation time of organs, thereby addressing the time-limit problems of organ transplant operations, especially in situations requiring long-distance transport. Thus, this method can increase the success rate of transplants and provide surgeons more leeway in arranging surgical procedures.

In organ transplantation surgery, artificial hibernation technology can help the recipient enter a temporary hibernation mode to reduce the activity of their immune system. This can reduce the danger of immune rejection during the surgery and decrease the amount of immunosuppressants needed after the operation, facilitating post-transplant functional recovery and maintaining organ viability. Artificial hibernation techniques such as TH have been proven to greatly reduce rejection reactions in kidney transplant recipients (Malinoski et al., 2023) and also ease oxidative stress, restrict cellular apoptosis, and reversibly inhibit immune activation, presenting new ways to deal with challenges related to kidney transplantation and IRI. Malinoski et al. (2019) examined the 1-year graft survival rates among 370 recipients of solid-organ transplants and found that the survival rate among recipients whose donors had been exposed to MH (34–35°C) before donation was 97%, while it was 93% among those with normothermic donors (36.5–37.5°C). This finding indicates that MH in donors can safely reduce the occurrence of delayed graft function in kidney transplant recipients without adversely affecting donor physiology or extra-renal graft survival. Specifically for kidney transplants from mildly hypothermic donors, the 1-year graft survival rate increased significantly, enhancing long-term patient outcomes. In general, the use of artificial hibernation technology, which entails maintaining organs at lower temperatures and diminishing immune responses, has been demonstrated to improve transplant outcomes by raising survival rates and lessening the risk of postoperative rejection.

## Applications of Artificial Hibernation Technology in the Management and Surgical Treatment of Central Nervous System Disorders

### Craniocerebral injury

Craniocerebral injury refers to a state where the structure and function of brain tissue are impaired by external physical forces acting on the head (Long et al., 2025). On the basis of their injury mechanisms, pathophysiological traits, and clinical presentations, craniocerebral injuries can be categorized into different types, and the classification mainly depends on the specific injury mechanisms as well as the pathological aspects that can distinguish closed and open injuries (Naeimi et al., 2024): closed injuries usually occur because of external forces causing compression or bruising of brain tissue while open injuries are marked by skull fractures and the exposure of brain tissue (Lee et al., 2023).

For patients undergoing craniocerebral injury surgery, the use of artificial hibernation technology could effectively cut down tissue oxygen requirement, protect the BBB, hold back the discharge of harmful substances, and lessen secondary brain harm. Moreover, in these patients, TH may alleviate neurological dysfunction after injury and reduce the ICP, making this a crucial area of study in modern neuroprotection research. This technology can also create a more favorable recovery environment for edema and epileptic seizures induced by brain injuries (Kobata, 2024). Therefore, hypothermia therapy should be regarded as a main treatment method for dealing with elevated ICP in patients with TBI who had undergone craniotomy and decompression. Li et al. (2009) stated that in a study involving 22 children with serious TBI, the 12 children receiving moderate hypothermia showed constantly lower ICP than the control group at all observation time points. In addition, the cerebrospinal fluid levels of neuron-specific enolase, S-100 protein, and brain-specific creatine kinase were much lower in the hypothermia group than in the normothermia group. In addition, at the end of the monitoring period, pH levels and electrolyte balance were within normal limits. These findings demonstrated that moderate hypothermia provides neuroprotective advantages for pediatric patients with severe TBI. The proper use of artificial hibernation technology in disease treatment can reduce the harmful effects of insufficient supply. Ridley et al. (2021) conducted a study on the energy consumption of 40 patients with TBI and observed that those who received prophylactic hypothermia treatment showed 20% less energy expenditure than the normothermic patients; the hypothermia group also showed higher gastric residual volumes, more frequent use of prokinetic agents, and energy delivery more closely matching measured energy requirements. These findings suggested that prophylactic hypothermia treatment may offer protective benefits to TBI patients while optimizing energy management. MH treatment has also been proven to stop ischemic and traumatic neuronal death, and research has confirmed its safety and effectiveness for treating traumatic SCI.

Artificial hibernation technology significantly lessens postoperative complications and mortality linked to craniocerebral injuries by slowing down brain metabolism, protecting the BBB, and alleviating inflammatory reactions, thereby improving patient outcomes.

### Extensive cerebral infarction and cerebral hemorrhage

#### Application of artificial hibernation technology in the management of extensive cerebral infarction and surgical procedures

Extensive cerebral infarction is the term used to describe the widespread ischemic necrosis of brain tissue ensuing from the sudden blockage of major cerebral arteries like the middle cerebral artery (Kobeissi et al., 2024). Extensive cerebral infarction is usually linked to severe neurological impairments and a high rate of disability. It is characterized by rapidly worsening neurological symptoms, a substantial risk of cerebral edema, and a poor prognosis. In this situation, artificial hibernation technology can provide a protective setting for brain tissue by methodically lowering the patient’s core body temperature and metabolic rate and thereby alleviating potential secondary injuries (Hanifa et al., 2024). During the treatment process, the main role of artificial hibernation technology is to reduce cerebral metabolism and lessen the oxygen consumption and energy needs of brain tissue, thereby reducing further harm to ischemic areas. Moreover, the low-temperature state can ease inflammatory reactions and diminish cell apoptosis (Yan et al., 2025). The combination of MH therapy and intravenous thrombolysis can significantly reduce the levels of IL-1β, IL-6, C-reactive protein, intracellular adhesion molecule-1, and matrix metalloproteinase-2 in patients with acute cerebral infarction, consequently limiting inflammatory responses (Li et al., 2022).

The use of artificial hibernation methods before, during, and after surgical operations can provide various forms of brain protection. The current therapeutic timeframe for brain infarction is highly restricted, so the preoperative use of artificial hibernation could stretch this timeframe for clot-busting or clot-removing processes, thereby enhancing treatment outcomes (Hassanipour et al., 2020). A European multicenter randomized phase III clinical trial by van der Worp et al. (2014) showed that moderate hypothermia (32–35°C) considerably reduced the expression of TNF-α in brain tissue and decreased the histological injury score, irrespective of the sedative utilized (propofol or dexmedetomidine). This finding implies that hypothermia performs neuroprotective functions by weakening inflammatory reactions. Moreover, the combination of hypothermia and dexmedetomidine simultaneously alleviate lung and kidney injuries, indicating that the overall anti-inflammatory impact may indirectly strengthen brain protection through systemic methods, such as curbing cytokine storms. Battaglini et al. (2022) proved that artificial hibernation induced by a combination of moderate hypothermia (32–35°C) and dexmedetomidine administration significantly reduced the expression of TNF-α in brain tissue and reduced the brain injury score. Bai et al. (2024) evaluated 44 patients with large-area cerebral infarction who had undergone successful reperfusion after endovascular treatment. Their study compared the results between patients receiving targeted temperature management (TTM) and those who did not, and their results showed that the percentage of patients attaining a favorable outcome at 90 days after treatment was much higher in the TTM group (46.7%) than in the non-TTM group (27.6%). This result indicates the possible efficacy of TTM for enhancing outcomes in patients with large-area infarction after reperfusion and suggests that the use of artificial hibernation technology after surgery can improve patient prognosis, reduce the risk of malignant brain edema, decrease the need for decompressive craniectomy (DC), and boost neural plasticity (Suerte et al., 2024).

In cases wherein traditional treatment methods yielded less-than-ideal results, the use of artificial hibernation technology to ameliorate large-area cerebral infarction showed remarkable effectiveness. Moreover, this technology generally offered a vital protective measure in the surgical management of widespread cerebral infarction since it reduced brain damage during the operation and improved postoperative recovery (Zhang et al., 2024a, b). Thus, with future advancements in this technology, its application potential in the field of neurosurgery can be expected to expand.

#### Application and significance of artificial hibernation technology in the management and surgical treatment of cerebral hemorrhage

Cerebral hemorrhage is a form of non-traumatic bleeding that occurs inside the brain due to the rupture of blood vessels in the brain and is regarded as an acute cerebrovascular occurrence. It is characterized by the entry of blood into the brain tissue to form a hematoma, which compresses the surrounding structures to cause neurological malfunction (Wu et al., 2025). Depending on the underlying cause, cerebral hemorrhage can be classified as primary intracerebral hemorrhage, which is mainly caused by hypertensive arteriosclerosis or cerebral amyloid angiopathy and often observed in deep brain areas such as the basal ganglia and thalamus, and secondary intracerebral hemorrhage, which can be triggered by vascular abnormalities, coagulation problems, tumors, or drug-related factors. Artificial hibernation technology can also play a vital role in treating cerebral hemorrhage during the acute stage. By reducing the patient’s body temperature, artificial hibernation can considerably lessen the metabolic requirements of brain tissue and thereby reduce the energy expenditure of brain cells and minimize the cell injury caused by lack of oxygen and blood flow (Lavinio et al., 2023). Moreover, by regulating body temperature, artificial hibernation technology can offer doctors more operation time while curbing inflammatory reactions and the death of nerve cells (Cadena and Rincon, 2024). Artificial hibernation can also assist in controlling the ICP. A cerebral hemorrhage is frequently followed by an increase in the ICP, which leads to further compression of brain tissue and even causes serious complications such as brain herniation (Myftiu et al., 2024). Thus, appropriate control of the ICP and facilitate subsequent surgical treatment or other interventions.

Research has shown that promptly applying artificial hibernation technology after cerebral hemorrhage can considerably increase patient survival rates and reduce the occurrence of long-term neurological dysfunction (Gouvea et al., 2023). Moreover, in a group of 60 patients diagnosed with low-grade aneurysmal subarachnoid hemorrhage, TTM, which entailed maintaining a temperature between 33–35°C for 3–5 days yielded much better prognoses than standard care, emphasizing the effectiveness and safety of TTM in improving neurological outcomes for patients with low-grade aneurysmal subarachnoid hemorrhage. Subsequently, Liu et al. (2024b) conducted a secondary analysis of 43 patients with low-grade aneurysmal subarachnoid hemorrhage who had undergone DC and compared the results obtained using DC alone with those obtained by combining DC and TTM (TTM-DC). Their results showed that the TTM-DC group, in which the core temperature was maintained at 34–35°C for at least 72 hours showed a far higher rate of favorable functional outcomes at 3 months (61%) in contrast to the DC-alone group (22%). Moreover, the TTM-DC group showed more significant improvements in Glasgow Coma Scale scores at discharge, indicating that the combination of TTM and DC may boost short-term neurological function in patients with low-grade aneurysmal subarachnoid hemorrhage and should be considered as a possible main treatment choice for these patients. Moreover, during the surgical handling of intracerebral hemorrhage, artificial hibernation technology is of vital importance, because first, by inducing hypothermia, artificial hibernation can greatly reduce blood loss during surgery since the vasoconstriction linked to a hypothermic state reduces bleeding at the surgical site and thereby enhances the safety and accuracy of the operation (Kobata et al., 2024). Moreover, induced hibernation can further lower the patient’s metabolic and heart rates to reduce the required amounts of anesthetic agents and minimize anesthesia-related risks. Patients in a hypothermic state show reduced reactions to pain and stress, ensuring better physiological stability throughout the surgical process.

These findings clarify the substantial application value of artificial hibernation technology in the management and surgical operations for hemorrhagic states, highlighting the improved treatment results and the quality of life for patients (**Table 2**).

**Table 2:** Comparison of the therapeutic effects of artificial hibernation on extensive cerebral infarction and cerebral hemorrhage

Disease	Therapeutic effects of artificial hibernation
Extensive cerebral infarction	Reduce brain metabolism and minimize further damage to ischemic areas.
	Reduce inflammatory responses and decrease cell apoptosis.
	Prolong the time window for thrombolysis or thrombectomy treatment and improve the therapeutic effects.
	Reduce brain damage during the operation and improve postoperative recovery.
Cerebral hemorrhage	Reduce the metabolic demands of brain tissue and minimize cell damage.
	Regulate body temperature to provide more time for surgery or other interventions.
	Intracranial pressure reduces further compression of brain tissue and complications.
	Control inflammatory responses and reduce nerve cell death.
	Reduce the amount of bleeding during the operation and enhance the safety of the surgery.
	Maintain the patient's physiological stability and reduce the dosage and risk of anesthetics.
	Promote postoperative recovery and reduce the incidence of complications.

### Neonatal hypoxic-ischemic encephalopathy

Neonatal hypoxic-ischemic encephalopathy (HIE) is a CNS condition caused by hypoxic-ischemic damage to brain tissue due to perinatal asphyxia (Cavanagh et al., 2025; Flyger et al., 2025; Shankaran et al., 2025). Common causes of neonatal HIE include placental abruption, nuchal cord, and difficult labor. The initial injury is characterized by hypoxia and the resultant collapse of brain cell energy metabolism (ATP depletion), which leads to sodium-potassium pump malfunction, cellular swelling, and lactic acidosis (Ananthan et al., 2025). The secondary injury that occurs after reperfusion is marked by an overproduction of free radicals, excitatory amino acids (such as glutamate), and inflammatory mediators (such as IL-6), which subsequently trigger neuronal apoptosis and necrosis (Chan et al., 2024).

Artificial hibernation technology has shown remarkable therapeutic efficacy in treating neonatal HIE, as proven by Collins et al. (2024). By reducing the body temperature, artificial hibernation technology can decrease the metabolic needs of brain tissue and attenuate the hypoxic harm to brain cells. Moderate hypothermia can efficiently hold back neuronal apoptosis, reduce inflammatory reactions, and maintain the integrity of the BBB (Faix et al., 2025). This is particularly vital for managing neonatal encephalopathy since the neonatal brain is not fully developed and has a low tolerance for hypoxia. Artificial hibernation could offer better protection to brain cells against oxidative stress damage by regulating neurotransmitter release and preventing free radical production (Lo et al., 2024). Moreover, in clinical situations, it is often combined with other therapeutic methods such as pharmacotherapy and hyperbaric oxygen therapy to optimize therapeutic outcomes. In cases of neonatal HIE, artificial hibernation technology also plays an important role in surgical treatment. During surgery, cerebral tissue may sustain additional damage due to IRI, and artificial hibernation can greatly reduce the cerebral metabolic rate during surgeries by decreasing core body temperature and thus lessening the oxygen and energy requirements of brain cells (Landucci et al., 2025). These effects protect the brain tissue from the effects of surgical trauma and widen the surgical time window, giving surgeons more sufficient operating time. Moreover, they reduce the occurrence of postoperative complications by suppressing the inflammatory response and oxidative stress during the operation. Thus, the use of artificial hibernation technology in complex pediatric neurosurgical procedures had been proven to significantly improve surgical outcomes and patient prognosis.

Huang et al. (2024) conducted a study evaluating the efficacy of TH in neonates with HIE. Their results showed a significantly lower incidence of abnormal MRI findings, moderate-to-severe cases, and watershed injury in the TH group than in the control group. Thus, TH can effectively reduce the occurrence of abnormal MRI findings and watershed injuries in neonates with mild HIE, without causing evident adverse reactions. These results imply that TH may offer neuroprotective benefits for neonates with HIE.

Nevertheless, the use of artificial hibernation in surgical operations requires more research and improvement to ensure its safety and effectiveness, so future research should focus on evaluating how artificial hibernation can be combined with other surgical technologies and identifying the best ways to apply this in various surgical situations.

### Post-cardiopulmonary resuscitation encephalopathy

Post-cardiopulmonary resuscitation encephalopathy refers to brain damage caused by overall ischemia and lack of oxygen following cardiopulmonary resuscitation. It is characterized by disturbances in consciousness and neurological impairments that can lead to a vegetative state or brain death (Goh et al., 2022). Artificial hibernation technology has been shown to play a crucial role in the treatment of brain disorders that occur after cardiopulmonary resuscitation. After such resuscitation, patients often experience severe brain injuries, which can be induced by numerous factors, including oxygen deficiency, ischemia, inflammatory reactions, and neuronal death. However, artificial hibernation can decrease body temperature and reduce metabolic rate, thereby lowering the energy consumption of brain tissue, suppressing neuronal excitability, weakening inflammatory responses, and ultimately reducing the severity of brain injury (Xia et al., 2024).

In clinical settings, artificial hibernation is typically induced through a combination of pharmacological substances and surface-cooling methods. Drugs such as barbiturates have been shown to suppress CNS activity, while surface-cooling approaches, including cold compresses and cooling blankets, effectively lower body temperature (Podsiadlo et al., 2024). Artificial hibernation technology can maintain body temperature between 32°C and 34°C, significantly reducing cerebral edema and neuronal apoptosis, and increasing patients’ chances of survival as well as neurocognitive and functional recovery (Hughes et al., 2025). Furthermore, artificial hibernation provides a valuable time window for subsequent neuroprotective measures, including the use of antioxidants, anti-inflammatory agents, and neurotrophic factors (Endo et al., 2024).

Staudacher et al. (2024) conducted a study to investigate whether TTM could enhance the neurological prognosis of patients following cardiac arrest. Among the 403 participants, 45.8% achieved favorable neurological outcomes, suggesting that a less sedative approach during TTM post-cardiac arrest may improve patient outcomes. Chen et al. (2024) examined the influence of TTM on patients with out-of-hospital cardiac arrest (OHCA) and found that TTM was associated with improved all-cause mortality. Specifically, levels of IL-6 and the IL-6/soluble IL-6 receptor complex were significantly lower in the TTM group compared to the control group. Additionally, both 90-day mortality and adverse neurological outcomes were reduced in the TTM group, indicating that TTM may enhance survival and neurological outcomes in patients undergoing resuscitation after OHCA by mitigating IL-6-induced pro-inflammatory responses. Shoji et al. (2024) conducted a study involving 1252 patients with OHCA who underwent extracorporeal cardiopulmonary resuscitation. Their findings indicated that the survival-to-discharge rate in the AH group remained stable regardless of the low-flow duration. Conversely, the survival-to-discharge rate in the non-AH group exhibited a declining trend as the low-flow duration increased. Interaction analysis revealed a significant interaction between low-flow duration and AH status concerning survival-to-discharge rates. Thus, OHCA patients presenting with a body temperature below 32°C and receiving extracorporeal cardiopulmonary resuscitation had relatively better survival outcomes, regardless of the low-flow duration. Therefore, early initiation of localized brain hypothermia can significantly enhance neurological outcomes and survival rates post-cardiac arrest, but careful management of electrolytes and renal function is essential.

Artificial hibernation technology shows great potential for treating brain disorders that occur after cardiopulmonary resuscitation. However, the potential risks and complications require careful consideration. For example, researchers have reported that low body temperature can lead to abnormal heart rhythms, issues with blood clotting, and an increased risk of infection. Therefore, when inducing artificial hibernation, it is essential to monitor the patient’s vital signs and adjust the treatment plan to address individual patient needs.

## Comprehensive Safety Evaluation

Although artificial hibernation technology has been used in clinical settings to protect neural tissues and organs by reducing metabolic rate and body temperature, its potential negative effects require consideration. Low temperatures may trigger adverse cardiovascular responses, such as arrhythmia and unstable blood pressure, which could endanger the patient’s vital signs (Cano et al., 2024). Hypothermia can also weaken the immune system, diminishing the patient’s ability to resist infections and increasing the risk of postoperative complications. In the nervous system, hypothermia can lead to a decrease in cerebral blood flow, potentially causing cerebral hypoxia and, in severe cases, neurological damage. Thus, despite the theoretical protective advantages of artificial hibernation technology, its potential adverse effects necessitate increased clinical caution and the implementation of appropriate preventive and therapeutic measures during the clinical application of this technology (Park et al., 2024).

In addition to the adverse reactions mentioned above, artificial hibernation technology may trigger a range of toxic reactions that could pose significant long-term health hazards to patients (Ran et al., 2024). A low-temperature setting is likely to disturb the chemical balance within and outside cells, with an imbalance between calcium and sodium ions potentially inducing cytotoxic reactions that can cause cellular malfunction or programmed cell death. Additionally, the metabolic rates of some sedatives and anesthetics decrease in cooler conditions, leading to higher drug levels and an increased risk of toxic reactions (Nakamura et al., 2024). Finally, artificial hibernation technology can also affect the repair and regenerative abilities of organs (Kamali et al., 2024), potentially resulting in longer postoperative recovery periods and, in severe cases, organ failure. Thus, although artificial hibernation technology shows protective advantages in certain situations, its possible adverse reactions necessitate comprehensive assessments and constant surveillance by clinicians to ensure patient safety and maximize treatment outcomes.

## Constraints and Challenges of Artificial Hibernation Technology

Artificial hibernation technology shows great potential in preserving neural tissues and organs, but its practical use was held back by several limitations. First, rodent models cannot fully represent humans, and the existing animal models have certain limitations. Second, the use of artificial hibernation technology in surgical procedures presents difficulties such as precisely controlling body temperature and reducing complications related to low-temperature states along with other technical problems. Therefore, future research should focus on improving the implementation of artificial hibernation to ensure its safety and effectiveness during surgery.

For instance, although hypothermia therapy has been shown to be effective in treating acute encephalopathy in pediatric patients, it also poses risks such as improper body temperature control and reperfusion injury during the rewarming process. Moreover, hypothermia can lead to systemic problems, including arrhythmia, cardiac arrest, and blood clotting disorders, which limit its use in complicated clinical situations (Imataka et al., 2023). Taccone et al. (2024) conducted a study involving 600 patients who had experienced OHCA and observed that the survival rates and rates of favorable neurological outcomes did not vary substantially between the 294 patients in the hypothermia treatment group and those in the normothermia group. However, the occurrence of arrhythmias was significantly higher in the hypothermia group. Their results suggested that maintaining the temperature at 33°C did not improve survival or neurological outcomes in patients with similar cardiac arrest characteristics. Additionally, metabolic inhibition in neural tissues under low-temperature conditions may weaken cellular functions, and low temperatures can disrupt cell signaling pathways and gene expression, potentially causing neuronal apoptosis or functional impairments. Furthermore, low-temperature conditions may reduce neuronal synaptic plasticity, affecting the long-term functional recovery of neural networks. Qiu et al. (2023) emphasized that in complex neural networks, low temperatures may hinder the self-repairing ability of neurons, and prolonged periods of hibernation caused by low temperatures could lead to irreparable neural damage. The clinical use of artificial hibernation technology also faces significant ethical hurdles.

To address the limitations of artificial hibernation technology, future research should focus on the following aspects: comprehensive evaluations of the mechanisms by which neurons regulate metabolism in low-temperature settings and the identification of novel approaches to protect neuronal functions; the creation of sophisticated and accurate body temperature control technologies to minimize the potential harm that low temperatures may inflict on neural tissues; and the establishment of large-scale clinical trials to evaluate the safety and effectiveness of artificial hibernation technology in various medical situations.

## Progress in Clinical Translation

Although fundamental research in artificial hibernation technology has made progress, some obstacles to clinical utilization remain unresolved (**Box 1**).

Box 1: Progress in clinical translation of the researches in artificial hibernation technology

**(1) Global distribution of clinical trials**

At present, the global distribution of clinical trials for this technology is uneven, and is primarily centered in regions such as North America, Europe, and Asia. Moreover, most of these trials have addressed areas such as TBI, SCI, heart surgery, and organ transplantation (Lu et al., 2021; Park et al., 2021; Ho, 2025). Among these, the majority were phase II trials, which mainly focused on evaluating safety and initial efficacy, suggesting that this technology holds a promising outlook for application. More recently, the number of clinical trials associated with artificial hibernation registered on ClinicalTrials.gov has been growing annually, particularly in emerging research domains such as neonatal HIE and acute liver failure (Artru et al., 2024; Kong and Lu, 2024).

**(2) Approval from the US Food and Drug Administration (FDA)**

Some hibernation medications have obtained conditional approval from the FDA, mainly for specific heart surgeries. Their mechanisms of action primarily target the hypothalamic thermoregulatory center, regulating body temperature to mitigate cardiac IRI and thereby reducing surgery-related complications while yielding neuroprotective effects. Consequently, the FDA's approval of these drugs signifies a crucial step forward in the clinical utilization of artificial hibernation-related technologies, particularly regarding their potential application in heart surgeries (Shen et al., 2024). Over the past several years, the number of drug patent applications in the field of artificial hibernation has shown a notable upward trend, and patent applications related to equipment have also surged rapidly. This reflects the growing attention given to minimally invasive surgeries and intelligent medical devices. Sub-temperature technology and CNS regulation technology offer new directions for the development of related equipment (Sakurai, 2022). Nevertheless, the relevant patents for artificial hibernation technology present substantial technical barriers to the development of related products and pose high risks in terms of research and legal costs. The complexity involved in applying artificial hibernation and the limitations imposed by related patents may slow the pace of its clinical transformation. Thus, overcoming these technical barriers and legal risks is an essential precondition for promoting the clinical transformation of artificial hibernation technology.

**(3) The financial support for the National Institutes of Health (NIH)**

The financial support for artificial hibernation research provided by the NIH has considerably increased, and some studies have focused on investigating the application of artificial hibernation in research areas including cardiopulmonary resuscitation and post-traumatic stress disorder. The research outcomes in these fields are anticipated to yield vital references for future clinical practice (Wright et al., 2021). The research and application of artificial hibernation technology have shown remarkable potential for clinical transformation in the realm of neuro-tissue and organ protection. The number of clinical trials in this area has significantly increased, particularly for studies related to acute nerve injury and organ transplantation. This trend indicates a growing interest and anticipation within the medical community regarding this field.

Although the US FDA has maintained a cautious stance during the approval process, it is still progressing steadily. The acknowledgment of breakthrough therapies represents an affirmation of artificial hibernation technology, highlighting its potential clinical value. With ample resources and support, these advancements have accelerated the transformation of research findings into clinical applications. Nonetheless, despite rapid development, artificial hibernation technology still faces numerous challenges. Ensuring safety and assessing long-term effects are pressing issues that need to be addressed. These challenges not only pertain to the practicality of the technology but also directly influence the safety and treatment outcomes for patients. Therefore, future research should place greater emphasis on establishing a comprehensive evaluation system to ensure the safety and efficacy of this technology in clinical applications.

## Limitations

With advancements in research on artificial hibernation technology and an increased understanding of its applications, its potential uses in the medical field have grown considerably. In addition to ongoing studies in areas such as organ transplantation, surgery, and emergency care, artificial hibernation may provide additional treatment options in fields like anti-aging therapies (Pinho et al., 2022), trauma management, interventions for neurodegenerative diseases, and cancer treatments, making it an innovative medical solution. However, despite its broad potential for preserving neural tissues and organs, artificial hibernation technology faces significant challenges related to technology, safety, and ethics, as well as individual differences in responses and complications resulting from long-term, low-temperature conditions. Therefore, ensuring experimental safety, implementing effective monitoring and management procedures, and evaluating the long-term physiological and psychological effects of this technology are critical concerns that require immediate attention. As the technology continues to advance, artificial hibernation is expected to provide substantial support for human survival in extreme environments.

## Conclusions and Perspective

The application of artificial torpor technology may represent a feasible solution to mitigate the harm of environmental radiation associated with life-sustaining systems (Shi et al., 2021). The torpor induced by low-temperature conditions has been proposed as a viable approach to facilitate long-term manned space missions (Fukunaga, 2020). Artificial torpor technology may also hold significance for individuals with rare diseases or specific genetic makeups, particularly some rare metabolic disorders such as mitochondrial diseases, where patients exhibit abnormal energy metabolism (Protasoni and Zeviani, 2021). In these individuals, artificial torpor technology may reduce the metabolic rate by lowering body temperature, thereby decreasing the buildup of toxic metabolites. Moreover, in certain hereditary neurodegenerative diseases, such as Huntington’s disease or specific hereditary ataxias (Wilke et al., 2023), low temperatures may delay the death of nerve cells, providing time for gene therapy or stem-cell therapy. Additionally, for some hereditary periodic fever syndromes, such as familial Mediterranean fever (Tufan and Lachmann, 2020), torpor can suppress inflammatory responses and alleviate symptoms, making artificial torpor a potential treatment option for these conditions.

The unique aspect of this review is its systematic integration of advancements in neuroscience, organ transplantation, and critical care medicine. This is the first review to provide an in-depth examination of the unique multi-organ cooperative protective mechanisms of artificial hibernation while directly addressing the challenges of clinical translation, including the development of inducing drugs, accurate temperature regulation technology, and management of reperfusion injury. This work thus bridges a significant gap in the field.

Industry experts believe that future research and application in this field should focus on improving the accuracy of the technical process and ensuring the safety of those involved. To enhance technical accuracy, more in-depth investigations are needed to clarify the physiological mechanisms underlying hibernation. Additionally, scientists should aim to identify ways to precisely regulate various aspects of the hibernation process, such as its initiation, duration, and termination, as even a minor error during these steps can lead to serious consequences for the hibernating individuals. Ensuring both the physical safety and mental well-being of hibernating individuals is of utmost importance. If the existing technical and ethical challenges are successfully addressed, the widespread use of artificial hibernation technology could provide new opportunities for human survival and development. Furthermore, in scenarios involving global catastrophes such as asteroid collisions or massive epidemics, artificial hibernation may allow part of humanity to endure the catastrophe by remaining in a dormant state until conditions improve. In the long run, if artificial hibernation technology is developed and utilized responsibly, it has the potential to enhance the future of humanity and help us overcome some of the most daunting challenges ahead.

## Data Availability

*Not applicable.*
